# Structural and Social Determinants of Health in Asthma in Developed Economies: a Scoping Review of Literature Published Between 2014 and 2019

**DOI:** 10.1007/s11882-020-0899-6

**Published:** 2020-02-06

**Authors:** Kathryn Sullivan, Neeta Thakur

**Affiliations:** Department of Medicine, Zuckerberg San Francisco General Hospital, University of California San Francisco, San Francisco, CA USA

**Keywords:** Asthma, Health disparities, Social determinants of health, Structural determinants of health, Policy, Socioeconomic status, Race, Ethnicity, Racism, Discrimination, Housing, Neighborhood, Greenspace, Food insecurity, Nutrition, Psychosocial stress, Community violence

## Abstract

**Purpose of Review:**

Using the WHO Conceptual Framework for Action on the Social Determinants of Health, this review provides a discussion of recent epidemiologic, mechanistic, and intervention studies of structural and social determinants of health and asthma outcomes covering the period from 2014 to 2019.

**Recent Findings:**

A majority of studies and interventions to date focus on the intermediary determinants of health (e.g., housing), which as the name suggests, exist between the patient and the upstream structural determinants of health (e.g., housing policy). Race/ethnicity remains a profound social driver of asthma disparities with cumulative risk from many overlapping determinants. A growing number of studies on asthma are beginning to elucidate the underlying mechanisms that connect social determinants to human disease. Several effective interventions have been developed, though a need for large-scale policy research and innovation remains.

**Summary:**

Strong evidence supports the key role of the structural determinants, which generate social stratification and inequity, in the development and progression of asthma; yet, interventions in this realm are challenging to develop and therefore infrequent. Proximal, intermediary determinants have provided a natural starting point for interventions, though structural interventions have the most potential for major impact on asthma outcomes. Further research to investigate the interactive effect of multiple determinants, as well as intervention studies, specifically those that are cross-sector and propose innovative strategies to target structural determinants, are needed to address asthma morbidities, and more importantly, close the asthma disparity gap.

## Introduction

Asthma—the most common chronic disease of childhood—disproportionately impacts communities of color and low socioeconomic communities [1, 2]. It is associated with high healthcare costs [3] and responsible for a significant proportion of emergency room visits, missed school and work days, and poor quality of life [1, 4]. There is a one-and-half-fold increase in the prevalence of asthma in individuals of low socioeconomic status (SES) compared with those of high SES [2]. Blacks have 1.25 times the asthma prevalence (10.1%) and twice the mortality rates (22.7 deaths per million persons) of the US general population (national prevalence 7.9%, mortality rate 9.9 deaths per million persons) [1, 2].

As a multifactorial disease, asthma is influenced by biologic, social, and environmental exposures throughout the life course. Decades of studies have demonstrated that low SES and exposures connected to poverty are strongly associated with both developing asthma [5, 6] and subsequent poor asthma outcomes including pulmonary function [7], symptom burden [8, 9], and exacerbations [10]. More recent works have alluded to interactive effect across multiple social exposures and how these operate synergistically, leading to poor outcomes on the population level [11–14•]. These factors contribute to persistent disparities in asthma incidence, prevalence, and outcomes for US and global populations.

**Fig. 1:**
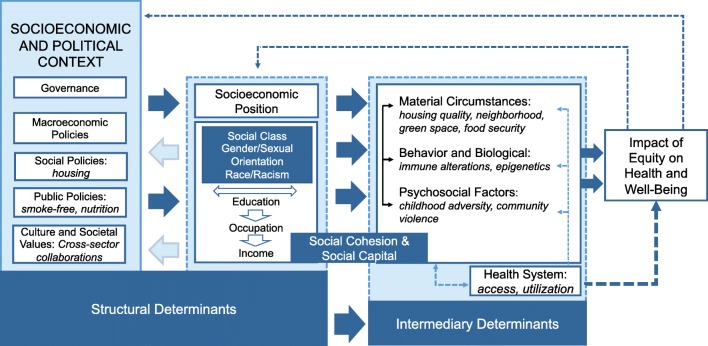
The level at which structural and social determinants affect asthma and contribute to poor asthma outcomes. Adapted from the WHO’s Conceptual Framework for the Social Determinants of Health [15]

In this review, we apply the World Health Organization (WHO) Conceptual Framework for Action on the Social Determinants of Health, created by the Commission on the Social Determinant of Health (CSDH), as demonstrated in Fig. 1 [15]. The WHO CSDH provides a foundation for understanding the significance of a rapidly growing number of studies exploring asthma disparities and points to where further study is needed. The framework differentiates how the socioeconomic and political contexts (e.g., government, policies, and cultures) manifest broadly as structural determinants (e.g., socioeconomic status, racism) which shape exposure to intermediary social determinants (e.g., housing conditions, psychosocial stressors) that ultimately create an individual’s unique social circumstances that influence behavior and interact with host/biologic factors. This approach allows us to address the immediate circumstances of living, while also considering the broader context that generates these circumstances. We highlight new evidence that further cement the role of social determinants in asthma, shed light on potential mechanisms for described associations, and report on interventions when available.

Given a wide range of economic conditions that impact health globally, this review focuses on research pertaining to nations classified by the United Nations as having developed economies [16] covering the period from 2014 to 2019. Although increasing literature is exploring the role of prenatal and birth exposures on outcomes in asthma, this review centers on exposures for individuals from infancy to adulthood.

## The Determinants

### Structural Determinants

The WHO CSDH recognizes a duality within the term social determinants of health, which includes both the social factors that influence health and the structural processes that determine the unequal distribution of these factors among groups resulting in social stratification of individuals within the socioeconomic-political context Fig. 1 [15].

#### Socioeconomic-Political Context

The socioeconomic-political context consists of broad structural and cultural aspects of society that are not measured on an individual level but have a profound effect on the organization of society, social position of individuals, and their health prospects. For instance, the 1930s historic US policy of “redlining,” which designated neighborhoods as “high” or “low” risk for housing loans, has had lasting impact by influencing neighborhood development, residential zoning, road and highway placement, and wealth distribution [17, 18]. As neighborhoods with higher proportions of people of color were often rated as “high” risk, or redlined, these communities are now disproportionately burdened by negative downstream effects [19, 20]. Although the political climate and government policies are foundational to health, there is a dearth of studies that specifically evaluate the effects of governance on asthma.

##### New Evidence

Housing code violations offer an aggregate-level examination of how poor home quality may cluster to impact asthma outcomes at the population level. In Cincinnati, housing code violations for asthma-related exposures (e.g., cockroaches and mold) correlated with population-level asthma morbidity independent of poverty [21]. Children who lived in census tracts in the highest quartile of housing code violations had 1.84 times more asthma-related hospitalizations compared with children in the lowest quartile (95%CI 1.36–2.48). This finding suggests how housing code violation density, or other administrative measures, could be used by hospitals or cities to identify key risk populations [21].

##### Current Interventions

For economically disadvantaged groups, housing remediation or relocation is particularly difficult given financial constraints, restrictions on improvements for rentals, shared exposures in multiunit housing, and vulnerability to eviction [22–25]. Housing voucher studies, such as Moving to Opportunity, showed mixed results; however, among those that did relocate, there were significant improvements in respiratory symptoms [22]. Newer policies, such as “Green Housing,” are focused on proactively implementing protective measures such as smoke-free housing policy, better pest-control practices, improved ventilation, and weatherization. In a case-control study of green vs. conventional housing in Boston, Massachusetts, children with asthma living in green housing had fewer asthma attacks (OR, 0.31; 95%CI 0.11–0.88), hospital visits due to asthma (OR, 0.24; 95%CI 0.06–0.88), and asthma-related school absences (OR, 0.21; 95%CI 0.06–0.74) compared with children living in conventional housing [26•]. A study in Chicago found similar benefits [27].

Another example of protective policy measures is the implementation of smoke-free policy for all public multifamily housing by the US Department of Housing and Urban Development [25]. In a study of 115 households in Colorado, these policies were effective at reduced smoking and secondhand smoke exposure reported by residents; effect was seen for respiratory complaints but not for asthma exacerbations [28]. A large study comparing asthma outcomes 3 years before and 3 years after indoor smoking legislation was introduced in 14 states and Washington, DC, found that smoke-free policy was associated with a 17% decrease in childhood emergency department visits for asthma exacerbation after controlling for secular trends, seasonality, male gender, Medicaid status, black race, and age (RR, 0.83; 95%CI 0.82–0.85) [29•].

Broad context-level interventions are not limited to governmental action, but can include public–private sector partnerships as illustrated by the AIR Louisville program, a collaboration among the Louisville Metro Government, a technology company, and a non-profit institute in Kentucky [30••]. This single-arm study used a geographic information system (GIS)-enabled inhaler sensors connected to a smartphone digital platform to monitor asthma medication use and symptom assessment for 497 children and adults with asthma. The data was used to tailor management and education for participants; use of the platform resulted in a 78% reduction in rescue inhaler use and a 48% improvement in symptom-free days. Authors also overlaid population-level medication use density data with maps of environmental features (e.g., air pollution) to identify hot spot areas for intervention. From their results, the cross-sector group made policy recommendations regarding tree canopy coverage, zoning laws for air pollution buffers around key institutions like schools and hospitals, a city-wide asthma notification system, and trucking routes. This study demonstrates how individual-level interventions can be leveraged for policy decisions and city planning.

#### Socioeconomic Position

Socioeconomic position (SEP) is one of the most well-studied contributors to asthma incidence and outcomes within developed economies [31, 32]. SEP encapsulates *social class* which relates to control over productive resources and *social stratification* which refers to differential access to opportunities. SEP consists of both resource-based measures (e.g., income) and prestige-based measures (e.g., access to knowledge or services via higher levels of education) [15]. The three most common indicators of SEP used in research are education, occupation, and income which can be measured at individual, household, and/or neighborhood levels. Comparisons across the literature are difficult as definitions are not equivalent. In this review, SEP and socioeconomic status (SES) are used interchangeably.

##### New Evidence

In the USA, disparities in healthcare access do not fully explain differences in outcomes by SES [31, 33]. New data from countries with universal health care systems further support the hypothesis that healthcare access alone does not account for the robust association between SES and asthma. A register-based cohort study in Sweden found incident asthma in children was associated with lower parental income in the first year of life and lower parental education levels regardless of child age [34]. Lower SES was also associated with asthma admissions in Canada [35] and England [36]. The role of occupation, with detrimental exposure risk often correlating with SES, should be studied further for asthma disparities [37].

##### Potential Mechanisms

There is conflicting evidence correlating SES with immune response; the type, timing, and duration of exposure to common environmental toxins (e.g., mold, dusts, pests, poor air quality) appear to delineate the direction of this association. In a cross-sectional study of SES and genome-wide mRNA expression in asthmatic children, downregulation of genes associated with a type I response and upregulation of those associated with a type II response were found in low SES children [38]. A second study supports this association between SES and type II response [39]. Prospective evaluation of immune responses to socioeconomic exposures among people without asthma is needed to delineate if this pathway is causative.

Effects of SES may be more broadly imprinted across the genome. Global DNA methylation, a marker of biologic age, was higher for low SES children with persistent asthma compared with higher SES children; however, these results were limited to black children [40]. A study by Galanter et al. found that among Latinx children of diverse sub-ethnicities with and without asthma, some but not all methylation differences could be explained by genetic ancestry, suggesting a mechanistic pathway for socioenvironmental stressors to increase disease susceptibility [41].

##### Current Interventions

A majority of interventions aimed at SES and asthma target multiple intermediary determinants of health, described elsewhere in this review, that coincide with low SES. Multilevel interventions to address adverse circumstances due to low SES (e.g., education, access to medications, home environmental interventions) have proven to be effective for asthma in a variety of communities [42•, 43–46]. Several of these interventions use the community health worker (CHW) model, which engages non-licensed lay persons, generally from the target community, to serve as a liaison between the health system and community. CHWs typically perform home visits for education, social support, self-management skill development, pest control, and care coordination [45••, 46]. Despite evidence for such programs, uptake is low [42•]. The next frontier is evaluating payment models [47] and other implementation strategies for these multicomponent, multilevel preventative programs.

#### Race/Ethnicity and Racism

Marked racial disparities in asthma incidence and outcomes exist both in the USA and other developed economies [48, 49]. Racial/ethnic boundaries are socially, not biologically, defined. Increasing evidence supports the notion that socioenvironmental exposures differentially distributed across race and ethnic groups, including SES and experiences of racism, drive these differences rather than shared genetic ancestry [50].

##### New Evidence

Risk factors for asthma such as SES are differentially experienced across racial/ethnic groups in the USA, yet asthma disparities persist even when SES is equivalent for minority groups. In the 2003–2004 National Survey of Children’s Health, higher SES was associated with lower incident childhood asthma for non-Hispanic white and black families compared with low SES families; however, the magnitude of the effect was smaller for black families [51]. The authors attribute this finding to the theory of Minorities’ Diminished Return, which posits that minority groups obtain a smaller health gain from equivalent improvements in socioeconomic or psychologic factors due to the daily structural barriers they face [52].

Asthma disparities may develop via racial/ethnic discrimination, independent of differential access to services and increased exposure to risk factors for minority populations. Building on work from the Black Women’s Health Study that demonstrated racism was associated with incident asthma in adult women [53], a case-control study found that black youth who reported higher levels of perceived discrimination had increased odds of having asthma (OR, 1.78; 95%CI 1.33–2.39) and of poor asthma control (OR, 1.97; 95%CI 1.42–2.76) [54••]. Discrimination was not associated with asthma for Latinx children in the study; rather, an interaction with SES was observed based on Latinx sub-ethnicity. Discrimination increased odds of asthma for Mexican American children with low SES and for other Latinx youth with high SES; these findings allude to the complex relationship between race/ethnicity and social status among different groups.

Lastly, asthma prevalence across Latinx subgroups varies widely [1]; however, the role of immigration and acculturation appears to affect groups similarly. The Genes-environments and Admixture in Latino Americans (GALA II) study, a large case-control study across 5 urban sites in the USA, found that greater levels of acculturation (defined by country of birth, generation in the USA, and language preference) were consistently associated with higher odds of asthma, extending the findings of acculturation as a risk factor for asthma for Mexican Americans [55, 56] to other Latinx subgroups in the USA [57•].

##### Potential Mechanisms

While several hypotheses may explain the racial disparities in asthma, growing evidence supports the idea that socioenvironmental factors—racially distributed due to decades of structural racism—rather than race-based or biologic differences themselves, are the driver of these disparities [58, 59]. Beck et al. attempted to elucidate what factors were responsible for observed differences in asthma-related admission rates between black and non-Hispanic white children in a prospective cohort [60••]. They found that socioeconomic hardship explained 53% the observed disparity gap, and when combined with atopy status, environmental, disease management, and access variables, 80% of the gap was explained and the observed differences were no longer significant.

#### Gender Identity and Sexual Orientation

Gender and sexual minorities experience higher levels of stress due to external (homophobia, discrimination) and internal (fear, rejection, identity concealment) stressors resulting from gender identity and sexual orientation [61]. As described for other psychosocial stressors, several studies have demonstrated that sexual minority populations have higher prevalence of asthma, particularly for lesbian and bisexual women [62, 63]. We have limited to no studies examining the relationship between gender identity and asthma.

##### New Evidence

Sexual minority women (SMW), who identify as lesbian or bisexual, have a higher prevalence of asthma. A meta-analysis including 12 studies found a higher prevalence of asthma for all SMW when adjusted for age (OR, 1.44; 95%CI 1.27–1.64) [64]. Other cross-sectional studies demonstrate persistence of this association even after adjusting for higher smoking prevalence and obesity among SMW [65, 66, 67•]. In pooled data from the National Health and Nutrition Examination Survey from 2009 to 2014, prevalence differences in asthma for SMW existed only when sexual orientation was defined by sexual identity and recent sexual behavior, but not by lifetime sexual behavior, i.e., no differences seen for women who identify as heterosexual with a history of same sex behavior compared with women with no history of same sex behavior [67•]. The authors posit that these differences may be explained by higher levels of psychosocial stress experienced by minority populations, though mechanistic studies are needed [61].

#### Social Cohesion and Social Capital

Social cohesion and social capital are determinants that bridge the structural and intermediary determinants in the WHO CSDH model. Social cohesion addresses a sense of community and solidarity between individuals that is important for the productive functioning of society and therefore health. Social capital is a complex debated term that centers on the notion of control over one’s circumstances [15].

##### New Evidence

Bellin et al. found that high levels of social cohesion and informal social control among caregivers of children with asthma modified the association between exposure to community violence and healthcare utilization [68•]. A material perspective on social capital is exemplified by a study examining the impact of financial constraints on family’s asthma management decision-making: US families with lower cost-sharing requirements on their insurance plans were less likely to delay care for physician visits (OR, 0.07; 95%CI 0.01–0.39) or ED visits (OR, 0.05; 95%CI 0.01–0.25) because of costs compared with families with a higher cost-sharing burden [69].

##### Current Interventions

The WHO CSDH stresses that the concept of social capital risks overemphasizing the role of an individual’s control rather than focus on the underlying structural forces responsible for generation of social capital [15]. The theory of “linking social capital” is thus valuable as it refers to interactions among people, groups, or institutions along gradients of institutionalized power [15, 70]. To provide effective care, health systems should be conscious of this dynamic by empowering communities to participate as stakeholders in design and implementation of clinical and research projects. Although it has not been explicitly examined, CHWs may be effective for asthma care because they are a link between institutions and individuals/communities. Typically, CHWs are from the local community, speak the same language, and are agile at navigating the local environment while also facilitating access to resources of clinics and hospitals; as such, CHWs may be a tool that increases social capital for individuals/communities by giving them access to power over their health [71, 72].

### Intermediary Determinants

The structural determinants of health dictate how intermediary determinants of health are dispersed and experienced across populations. As the intermediary determinants are closer in proximity to asthma-related outcomes, they may offer a more immediate opportunity for intervention. The focus on these interventions should not supersede movement toward more broad-based, comprehensive policy changes that address the underlying sources of inequity.

#### Material Circumstances: Housing Conditions, Neighborhoods, and Grennspace

##### Housing Conditions,

particularly indoor air pollution and microbial/pest allergen exposures, are a key determinant of asthma morbidity, particularly for poor urban populations [73].

##### New Evidence

In a national survey, poor housing quality was independently associated with asthma diagnosis (OR, 1.45; 95%CI 1.28–1.66) and ED visits (OR, 1.59; 95%CI 1.21–2.10), whereas home ownership was associated with lower odds of asthma-related ED visits (OR, 0.62; 95%CI 0.46–0.84) [74]. Housing differences did not fully explain a disparity in asthma morbidity between black and non-Hispanic white households.

##### Potential Mechanisms

Housing conditions clearly affect asthma through indoor microbial and non-microbial exposures [24, 73]. Some of the association between asthma and housing may be due to neighborhood factors described below.

##### Current Interventions

Interventions targeting housing exposures have significant potential to improve asthma outcomes for high-risk populations [75, 76]. A joint workshop among several different national agencies including the The National Institute of Allergy and Infectious Diseases has identified key research priorities for future indoor environmental interventions [77]. A Cochrane systematic review found moderate quality evidence that interventions to repair moisture and mold damage in houses and offices improved asthma outcomes including fewer acute care visits for children and decreased asthma symptoms for adults [78].

##### Neighborhoods,

the built environment, are an intersection of the social and physical environment, together shaping risks for asthma-related outcomes.

##### New Evidence

Living in “inner city” neighborhoods alone appears less likely to be a true intermediate determinate. In a national sample, Keet et al. found a higher prevalence of asthma in inner city neighborhoods, but this difference was not significant after adjusting for key demographic factors including race/ethnicity and SES [79]. Shmool et al. comprehensively examined aggregate, administrative measures of stress (e.g., health access, disorder, crime rates) and used factor analysis to determine patterns of social stress in New York City [14•]. They identified three patterns, one associated independently with higher asthma exacerbation rates and a second that was also associated with higher exacerbation rates, but only in neighborhoods with more traffic-related air pollution. Similarly, the urban–rural asthma divide may not hold up when accounting for socioeconomic and racial factors between regions [80].

##### Greenspace

has been increasingly recognized as beneficial for health. Greenspace refers to undeveloped areas with natural vegetation, though in urban environments it includes parks and street vegetation. The health benefits of greenspace may be related to a reduction in psychosocial stress (perhaps by providing area for exercise, increased opportunity for social interaction, emotional well-being) [81].

##### New Evidence

In the context of asthma, studies suggest that greenspace could increase exposure to microbial antigens that may impact immune system development, counteract environmental pollution of urban communities, and mediate family relationship stress [82–84]. Despite these hypotheses, there are inconsistent associations with greenspace and asthma [85–87]. Two meta-analyses demonstrated a non-significant trend toward reduced asthma incidence in areas with higher greenspace, though they are limited by heterogeneity [85, 87]. Factors that may account for the inconsistencies among studies include seasonality and the timing of the exposure (e.g., perhaps early life exposures are beneficial for immune system development, but after atopy has developed, allergen exposures could precipitate worse control). Furthermore, in different neighborhoods, greenspace could represent areas that are perceived to be unsafe [14•].

#### Material Circumstances: Food Insecurity and Nutrition

Food insecurity, or lack of adequate access to food, is common in the USA and is closely related to SES [88]. In the USA, food insecurity affected more than 20% of households with children from 2008 to 2012; more recently, this declined to 13.9% in 2018, reflecting economic stabilization after the 2008 recession [89].

##### New Evidence

A cross-sectional study in Hawaii found that adults with food insecurity had higher odds of asthma [90]. The Early Childhood Longitudinal Study–Kindergarten (ECLS-K), a prospective cohort study, found that this association starts in childhood, furthering the evidence for causality. Parent-reported food insecurity in the year before kindergarten or in second grade was associated with incident asthma by the third grade (OR, 1.18; 95%CI 1.17–1.20, and OR, 1.53; 95%CI 1.51–1.55, respectively) after controlling for BMI, parental depression, and demographic variables, though authors did not consider environmental risk factors (e.g., smoking) in their analyses [91, 92]. This evidence is consistent with findings from a prospective pediatric cohort study on hunger in Canada [88, 93].

##### Potential Mechanism

Exactly how food insecurity may increase risk for asthma remains unclear—does poverty force tradeoffs between medications and food [94], does the physiologic and psychologic stress of hunger drive inflammation, and/or does a nutritional component influence the pathogenesis [95, 96]? One study started to address these mechanistic questions: increased food diversity in the first year of life was associated with a dose-dependent decrease in asthma prevalence in a birth cohort study across five European countries [97]. Given that the social and environmental factors that contribute to obesity may also lead to food insecurity, nutritional insufficiency should also be considered when exploring the connection between obesity and asthma [98, 99].

##### Interventions

Early studies demonstrate some improvement in asthma morbidity with interventions that target food insecurity. A study of children on Medicaid receiving Supplemental Nutrition Assistance Program (SNAP) benefits found that across similarly low-income households, the larger quantity of household SNAP benefits was associated with a reduction in ED claims for asthma [100].

##### Special Considerations: Sugar-Sweetened Beverages

Sugar-sweetened beverages (SSBs) are a nutritional component that has received increasing attention over the last decade. SSBs are associated with asthma prevalence and morbidity, independent of obesity, in children and adults in cross-sectional and prospective studies [101–108]. In the prospective Framingham Offspring Cohort study, the consumption of SSBs with excess fructose content was associated with a dose-dependent increase in asthma risk for adults (HR, 1.89; 95%CI 1.36–2.62 for 5–7 servings/week) when adjusted for demographics, BMI, smoking, and type II diabetes [102••]. This finding was limited to beverages with a high fructose to glucose ratio (e.g., sodas high in fructose corn syrup and apple juice), but not for beverages without excess free fructose (e.g., diet soda and orange juice) [102••]. This difference may relate to intestinal formation of advanced glycation end products which may trigger pulmonary inflammation via RAGE receptors [109, 110]. These findings, if confirmed in further prospective studies and trials, would be particularly amenable to policy interventions for dietary guidelines, school lunch nutritional requirements, and food assistance programs.

##### Special Considerations: Vitamin D

Vitamin D insufficiency, higher in black populations [111], has been associated with asthma prevalence and morbidity; however, a causal mechanism remains controversial. Multiple studies support a role for vitamin D via its anti-oxidant and immunomodulatory effects [112–116]. Two meta-analyses of randomized clinical trials demonstrate that vitamin D supplementation reduced the rate of asthma exacerbations requiring systemic corticosteroids in both children and adults in ethnically diverse populations [117•], [118].

#### Psychosocial Stress: Childhood Adversity and Community Violence

The high co-occurrence of psychiatric disease and asthma suggests there may be a link between an individual’s psychologic health and asthma [119–121]. Now, it is increasingly understood that beyond psychiatric disease, certain psychosocial stressors may increase risk of or worsen asthma.

##### New Evidence

Adverse childhood experiences (ACEs) are a subset of psychosocial stressors which include measures of child abuse, neglect, and household dysfunction that have been associated with a variety of health outcomes [122]. Since the landmark ACE Study in 1998, an exponential number of studies have linked ACEs to poor health, including asthma prevalence in children and adults [123••, 124, 125]. In the National Survey of Children’s Health, a dose-dependent relationship was observed between ACEs and asthma prevalence in that children with at least five ACEs had increased odds of asthma (OR,1.61; 95%CI 1.15–2.26) in fully adjusted models that accounted for household smoking, prematurity, neighborhood, and demographics [123••]. Similar findings were obtained for adults in a national survey [124, 126]. Prospective studies are needed to assess the relationship between ACEs and asthma with more granularity.

Similar to other stressors, the effect of community violence on asthma outcomes likely manifests through the same dysregulation of the biological response to stress [127] and through indirect mechanisms (e.g., parental smoking, depression, medication adherence, or time indoors due to safety concerns) [128]. Multiple studies have built upon earlier work demonstrating an association between asthma and community violence across the age spectrum [108, 128–131]. Census tract level violent crime in Cincinnati was associated with increased ED utilization rates for children with asthma after adjusting for tract level rates of poverty, unemployment, substandard housing, and traffic exposure [132•]. Again, in a prospective study, Bellin et al. found that caregivers’ exposure to community violence predicted healthcare utilization for their children’s asthma. As reported above, this finding was not observed among children with caregivers who had higher levels of measured informal social control and social cohesion [68•], demonstrating potential resilience factors to target for future study.

##### Potential Mechanisms

There is an increasing body of literature demonstrating that psychosocial stressors cause *weathering* across biological systems that may progress to negative long-term health outcomes [133, 134]. These alterations lead to poor health through several pathways including dysregulated neuro-endocrine-immune activation and/or autonomic nervous system functioning—known as allostatic load [135, 136]; epigenetic modification [137]; alteration of the body’s microbiome [127, 138]. In a cross-sectional study of Puerto Rican children, Brehm et al. analyzed bronchodilator responses and stress levels [139]. Children who were “highly stressed” or had mothers who were “highly stressed” had a significantly lower bronchodilator response than children without personal or maternal high stress. Across several pediatric cohorts, the authors replicated this reduction in bronchodilator response in presence of high stress (total n = 2741). They found that the reduction in bronchodilator response was also associated a polymorphism of the ADCYAP1R1 gene, which has been linked to post-traumatic stress disorder and anxiety (*p*-for-interaction with stress = 0.01). This polymorphism was associated with β2-adrenergic receptor expression in T lymphocytes [139]. In the same study population, Chen et al. found that increased methylation of this gene, ADCYAP1R1, was associated with poorly controlled asthma among children who were also exposed to community violence [140].

##### Potential Interventions

Reducing exposure to psychosocial stressors and improving response to them are potential targets for intervention. A randomized clinical trial of Multisystemic Therapy—an intensive family and community-based approach—found that the intervention significantly improved lung function, adherence, and control for black adolescents with poorly controlled asthma [141] consistent with other evidence supporting family therapy for health [142]. Early data suggests other interventions to improve coping, such as mindfulness-based stress reduction, may be beneficial [143, 144]. Technological interventions may provide another means of behavioral support that could be less resource intensive than in-person therapy [145, 146]. Novel, real-time methodologies such as ecologic momentary assessment delivered via smartphones may be a useful tool for future prospective studies assessing the utility of interventions aimed at reducing psychosocial stress [147–149].

## Conclusion

This large body of evidence supports a fundamental connection between the structural and social aspects of health and asthma morbidity across one’s lifetime. It is essential that these factors are considered when developing asthma prevention and treatment programs. Substantial improvements for asthma outcomes will not be made without addressing the underlying societal processes that have created large and persistent disparities in asthma outcomes.
